# Comparative Evaluation of the Antimicrobial Efficacy of Endodontic Sealers Against Staphylococcus aureus and Streptococcus mutans: An In Vitro Study

**DOI:** 10.7759/cureus.80435

**Published:** 2025-03-11

**Authors:** Pranav Patil, Ana Gupta, Komal Kishlay, Sneha Rathaur, Neha Vaidya, Manish Sharma, Seema Gupta

**Affiliations:** 1 Department of Conservative Dentistry and Endodontics, Bharati Vidyapeeth Dental College and Hospital, Sangli, IND; 2 Department of Conservative Dentistry and Endodontics, Institute of Dental Sciences, Jammu, IND; 3 Department of Prosthodontics, Sarjug Dental College and Hospital, Darbhanga, IND; 4 Department of Conservative Dentistry and Endodontics, Rungta College of Dental Sciences, Bhilai, IND; 5 Department of Prosthodontics, Revive Dental Care, Bengaluru, IND; 6 Department of Oral Pathology, Jawahar Medical Foundations Annasaheb Chudaman Patil Dental College, Dhule, IND; 7 Department of Orthodontics, Kothiwal Dental College and Research Centre, Moradabad, IND

**Keywords:** inhibition, root canal, sealers, staphylococcus aureus, streptococcus mutans

## Abstract

Introduction: Endodontic therapy aims to eliminate microbial infections from the root canal system and prevent reinfection, thus ensuring periapical tissue healing. Persistent bacterial contamination, particularly from *Staphylococcus aureus* and *Streptococcus mutans*, is a major cause of endodontic treatment failure. Owing to the complexity of the root canal anatomy, conventional mechanical and chemical disinfection methods may be insufficient to eliminate bacteria. Endodontic sealers with antimicrobial properties play a crucial role in enhancing disinfection and preventing bacterial re-colonization. This study aimed to evaluate and compare the antimicrobial efficacy of three commonly used endodontic sealers, AH Plus (Dentsply Maillefer, Ballaigues, Switzerland), Zinc Oxide Eugenol (ZOE; Pyrax Cavibond, Roorkee, India), and Tubli Seal (SybronEndo, Glendora, CA, USA), against *Staphylococcus aureus* and *Streptococcus mutans* using the agar diffusion method.

Materials and methods: This in vitro study was conducted at the Department of Conservative Dentistry and Endodontics between May 2024 and October 2024. The antimicrobial properties of three endodontic sealers - AH Plus, ZOE sealer, and Tubli Seal - were tested against *Staphylococcus aureus* ATCC 25923 and *Streptococcus mutans* ATCC 700610. The bacterial suspensions were standardized to 1.5 × 10⁸ colony-forming units (CFU)/ml. Agar wells (6 mm diameter, 5 mm depth) were filled with freshly mixed sealers and inhibition zones were measured using a digital caliper at 24 hours and seven days. Statistical analysis was performed to compare the antibacterial efficacy of sealers over time.

Results: At 24 hours, AH Plus exhibited the highest antimicrobial activity against both bacterial species (p < 0.05), followed by the Tubli Seal and ZOE. By seven days, the ZOE sealer demonstrated the most potent antibacterial effect, surpassing AH Plus and Tubli Seal (p < 0.05). Both AH Plus and Tubli Seal exhibited a decline in the antibacterial activity over time. All the sealers were more effective against *Streptococcus mutans*.

Conclusion: Endodontic sealers exhibit time-dependent antibacterial properties. AH Plus is the most effective sealer in the early stages of treatment, providing strong initial bacterial suppression, whereas ZOE sealer demonstrated sustained antimicrobial activity over time. The Tubli Seal showed moderate efficacy. These findings emphasize the importance of sealer selection based on clinical scenarios.

## Introduction

Endodontic therapy primarily aims to eliminate microbial infections from the root canal system and prevent reinfection, thus ensuring the healing of periapical tissues. The persistence of microorganisms in the root canal system has been identified as the primary cause of endodontic treatment failure, leading to periradicular inflammation and post-treatment complications [1]. The complexity of root canal anatomy, including the presence of dentinal tubules, ramifications, deltas, and fins, creates an environment in which bacteria can evade mechanical instrumentation and antimicrobial irrigation. Consequently, the use of endodontic sealers with antimicrobial properties has been extensively investigated as an adjunct to conventional root canal disinfection methods [2].

Bacterial species such as *Staphylococcus aureus* and *Streptococcus mutans* are commonly associated with endodontic infections. *Staphylococcus aureus* is a facultative anaerobic gram-positive bacterium known for its biofilm-forming ability, which contributes to its resistance to antimicrobial agents [3,4]. It is implicated in persistent root canal infections and periapical lesions. *Streptococcus mutans*, another gram-positive facultative anaerobe, plays a significant role in dental caries and has been detected in infected root canals [4]. The presence of these bacterial species in the root canal system necessitates the use of endodontic materials that exhibit potent antimicrobial activity, to improve the success rate of endodontic treatment.

Endodontic sealers have multiple functions in root canal therapy, including sealing voids, filling lateral canals, and preventing microbial penetration. Apart from their physical sealing properties, an ideal endodontic sealer should possess antimicrobial activity to further reduce bacterial survival within the root canal [5]. Sealers with different compositions have been developed, and their antimicrobial effectiveness varies depending on their formulation and interactions with bacterial species. Among the commonly used endodontic sealers are AH Plus (Dentsply Maillefer, Ballaigues, Switzerland), Zinc Oxide Eugenol (ZOE; Pyrax Cavibond, Roorkee, India), and Tubli Seal (SybronEndo, Glendora, CA, USA), all of which possess distinct chemical properties that influence their antibacterial efficacy [6].

AH Plus is an epoxy resin-based sealer known for its superior sealing ability and prolonged antimicrobial activity owing to the release of formaldehyde during its polymerization process. Studies have reported that AH Plus exhibits significant antibacterial effects against *Staphylococcus aureus* and *Streptococcus mutans* in the initial phases of treatment [7]. ZOE-based sealers are widely used because of their antimicrobial properties derived from eugenol, which has been recognized for its bactericidal effects. However, the antibacterial effectiveness of ZOE diminishes over time as eugenol is released and diffuses away [8]. Tubli Seal, a polysiloxane-based sealer, has also been evaluated for its antimicrobial potential, although its effectiveness varies compared to other sealers [9].

This study aimed to compare the antimicrobial efficacy of three endodontic sealers (AH Plus, ZOE, and Tubli Seal) against *Staphylococcus aureus *and *Streptococcus mutans *using the agar diffusion method. Understanding the antimicrobial potential of these sealers could provide valuable insights into their role in improving endodontic treatment outcomes and preventing bacterial persistence in the root canal system.

## Materials and methods

Study design

This in vitro investigation was performed within the Department of Conservative and Endodontics at Bharati Vidyapeeth Dental College, Sangli, spanning from May 2024 to October 2024. The study did not involve human participants or human-derived tissues, obviating the need for ethical clearance, and was conducted in strict adherence to the principles outlined in the Declaration of Helsinki.

Sample size

Power analysis for sample size estimation was performed using G*Power software version 3.6.9 (Heinrich-Heine-Universität Düsseldorf, Düsseldorf, Germany) to achieve a statistical power of 80% with an alpha error of 5%. The calculation was based on an effect size of 0.41, as reported in a prior study by Shetty et al. [10], which examined the inhibition zones for *Staphylococcus aureus* using the endodontic sealers ZOE and AH Plus. These parameters were applied in an analysis of variance (ANOVA) fixed-effects omnibus one-way analysis considering three groups. The a priori computation yielded a total sample size of 60, evenly distributed into three experimental groups with 20 samples per group, thereby ensuring robust statistical validity for the study.

Methodology

**Figure 1:**
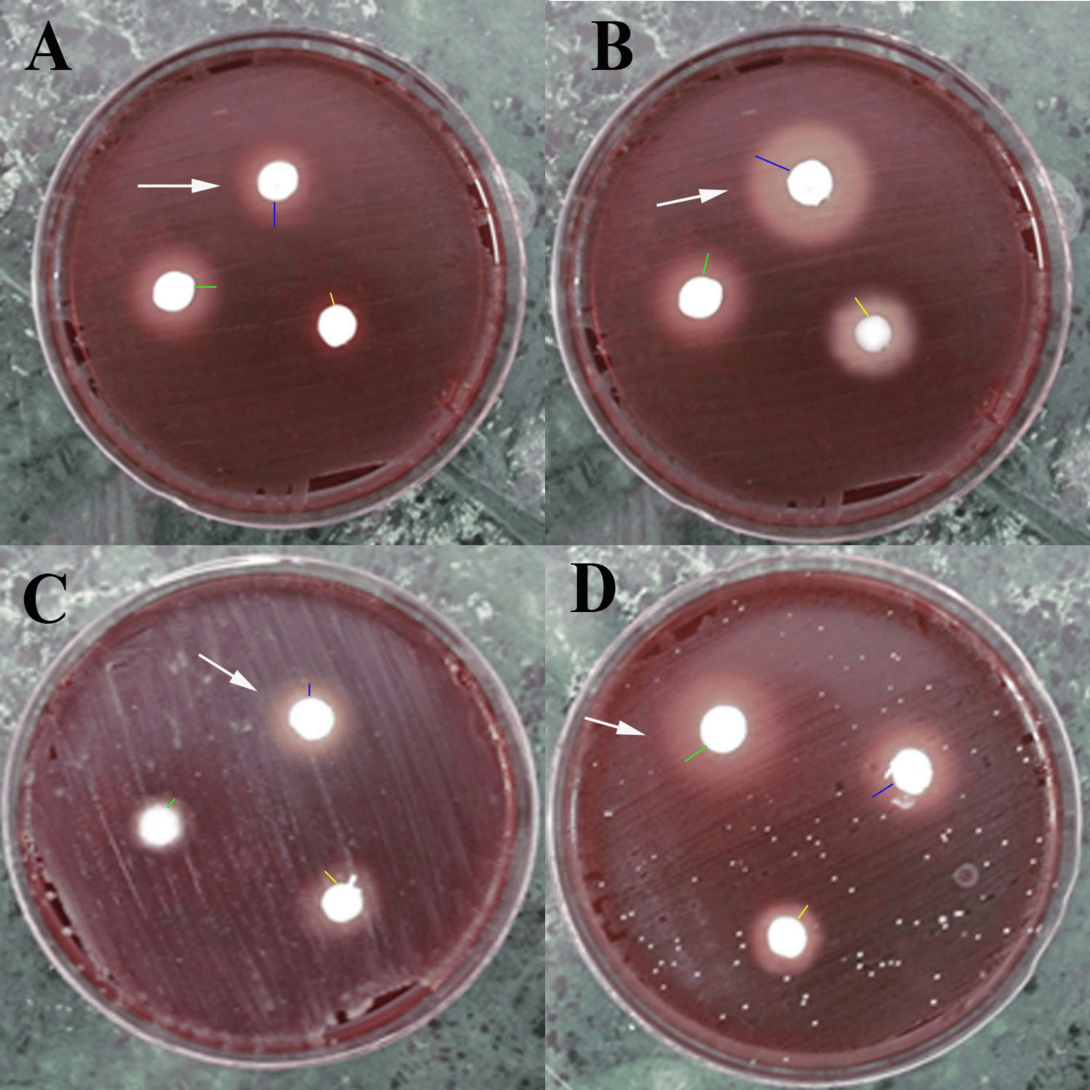
Microbial culture in agar plate (A) Inhibition zone for Staphylococcus aureus after 24 hours by ZOE, AH plus and Tubli Seal, (B) Inhibition zone for Staphylococcus aureus after seven days by ZOE, AH Plus and Tubli Seal, (C) Inhibition zone for Streptococcus mutans after 24 hours by ZOE, AH Plus and Tubli Seal, (D) Inhibition zone for Streptococcus mutans after seven days by ZOE, AH Plus and Tubli Seal. Zinc oxide eugenol (ZOE) (yellow line), AH plus (blue line) and Tubli Seal (green line).

This study evaluated the antibacterial properties of three endodontic sealers, which were divided into three groups with 20 samples in each group: ZOE sealer, AH Plus, and Tubli Seal. All sealers were prepared in accordance with the manufacturer’s instructions. The antibacterial efficacy of the sealers was tested against two bacterial strains, *Staphylococcus aureus* ATCC 25923 and *Streptococcus mutans* ATCC 700610 (both from American Type Culture Collection (ATCC), Rockville, MD, USA). The bacterial strains were cultured overnight in tryptic soy broth (Oxoid, Hampshire, UK) to obtain a fresh inoculum. The turbidity of the bacterial suspension was adjusted to 1.5 × 10^8^ colony-forming units (CFU)/mL using the 0.5 McFarland Baso 4 standard to ensure standardization of the inoculum density. All the tryptic soy agar Petri dishes supplemented with 5% sheep blood, hemin, and vitamin K were used as the growth media. Ten plates were designated for the 24-hour observation period, and the remaining 10 for the seven-day observation period (Figure 1).

Each agar plate contained three wells punched into the agar medium using a sterile puncher, with each well measuring 5 mm in depth and 6 mm in diameter. Sealers were prepared according to the manufacturer’s guidelines and immediately dispensed into the wells. The plates were kept at room temperature for two hours to allow the pre-diffusion of the antibacterial agents from the sealers into the agar medium. Following this pre-diffusion period, the plates were incubated under aerobic conditions at 37°C. The zone of inhibition around each well was measured by two independent examiners in millimeters in two perpendicular directions using a digital caliper. The average diameters of the inhibition zones were recorded to assess the antibacterial efficacy of the sealers. The measurements were repeated for both the 24-hour and seven-day incubation periods.

Statistical analysis

Statistical analyses were conducted using SPSS software version 23 (IBM Corp., Armonk, NY, USA). Normality of the data was assessed using the Kolmogorov-Smirnov test, and confirmed through Q-Q plots. Intraclass comparisons of inhibition diameters at different time points were analyzed using paired t-tests. To compare inhibition zones between microbial groups, independent t-tests were applied. Intergroup comparisons were performed using one-way ANOVA. Post-hoc analyses were conducted with Tukey’s multiple comparison test to identify significant differences between groups, with a significance level set at p < 0.05.

## Results

For both bacteria, ZOE showed a statistically significant (p = 0.001) greater inhibition zone at seven days than at 24 hours, which showed that its antibacterial activity increased over time, whereas AH Plus showed a statistically significant (p = 0.001) greater inhibition zone at 24 hours than at seven days, which showed that its antibacterial activity decreased over time. The Tubli Seal did not exhibit a significant difference in bacterial growth at either time interval (p = 0.301). These findings suggested that the antimicrobial effects of the tested sealers varied over time (Table 1).

**Table 1:** Intragroup comparison of mean inhibition zone at different time intervals by paired t test. ZOE: zinc oxide eugenol, Data is presented in form of mean ± standard deviation (SD), p-value < 0.05: significant.

Bacterial species	Sealer	Inhibition diameter (mm) after 24 hr	Inhibition diameter (mm) after 7 days	t-stats	p-value
		Mean ± SD	Mean ± SD		
Staphylococcus aureus	Group 1 (ZOE)	7.55 ± 1.74	13.88 ± 1.74	11.5	0.001*
	Group 2 (AH Plus)	15.11 ± 3.21	9.55 ± 3.21	5.47	0.001*
	Group 3 (Tubli Seal)	8.66 ± 3.80	7.41 ± 3.80	1.04	0.301
Streptococcus mutans	Group 1 (ZOE)	8.70 ± 1.98	17.00 ± 1.98	13.25	0.001*
	Group 2 (AH Plus)	19.22 ± 1.98	12.88 ± 1.98	10.12	0.001*
	Group 3 (Tubli Seal)	13.33 ± 2.34	10.00 ± 2.34	4.51	0.001*

At 24 hours, ZOE was equally effective against *Streptococcus mutans* and *Staphylococcus aureus* with no significant difference in antibacterial properties (p = 0.058), whereas AH Plus and Tubli Seal were more effective against *Streptococcus mutans* than against *Staphylococcus aureus* (p = 0.001). At seven days, all groups demonstrated a significant increase in antibacterial efficiency against *Streptococcus mutans*. These findings suggest that all groups were effective against *Streptococcus mutans* at prolonged time intervals, whereas ZOE was effective against both bacteria at short time intervals (Table 2).

**Table 2:** Comparison of mean inhibition zone between bacterial species by independent t test. ZOE: zinc oxide eugenol, Data is presented in form of mean ± standard deviation (SD), p-value < 0.05: significant.

Time interval	Groups	Staphylococcus aureus (Mean ± SD)	Streptococcus mutans (Mean ± SD)	t- test	p-value
Inhibition diameter (mm) after 24 hr	Group 1 (ZOE)	7.55 ± 1.74	8.70 ± 1.98	1.95	0.058
	Group 2 (AH Plus)	15.11 ± 3.21	19.22 ± 1.98	4.87	0.001*
	Group 3 (Tubli Seal)	8.66 ± 3.80	13.33 ± 2.34	4.56	0.001*
Inhibition diameter (mm) after 7 days	Group 1 (ZOE)	13.88 ± 1.74	17.00 ± 1.98	5.29	0.001*
	Group 2 (AH Plus)	9.55 ± 3.21	12.88 ± 1.98	3.94	0.003*
	Group 3 (Tubli Seal)	7.41 ± 3.80	10.00 ± 2.34	2.59	0.0138

At 24 hours, AH Plus exhibited the highest antibacterial efficiency, followed by Tubli Seal, and ZOE showed the lowest antibacterial efficiency (p = 0.001). At seven days, ZOE demonstrated the highest antibacterial efficiency, followed by AH Plus, and the lowest in Tubli Seal (p = 0.001). These findings suggest that AH Plus showed the best antibacterial properties for a short duration, whereas ZOE exhibited the best antibacterial properties for a long duration, suggesting its long-term effectiveness (Table 3).

**Table 3:** Intergroup comparison of mean inhibition zone by analysis of variance (ANOVA) test. ZOE: zinc oxide eugenol, Data is presented in form of mean ± standard deviation (SD), p-value < 0.05: significant.

Time interval	Bacterial species	Group 1 (ZOE)	Group 2 (AH Plus)	Group 3 (Tubli Seal)	F value	p-value
		Mean ± SD	Mean ± SD	Mean ± SD		
Inhibition diameter (mm) after 24 hr	Staphylococcus aureus	7.55 ± 1.74	15.11 ± 3.21	8.66 ± 3.80	36.0036	0.001*
	Streptococcus mutans	8.70 ± 1.98	19.22 ± 1.98	13.33 ± 2.34	125.25	0.001*
Inhibition diameter (mm) after 7 days	Staphylococcus aureus	13.88 ± 1.74	9.55 ± 3.21	7.41 ± 3.80	23.535	0.001*
	Streptococcus mutans	17.00 ± 1.98	12.88 ± 1.98	10.00 ± 2.34	55.77	0.001*

Pairwise comparisons revealed statistically significant differences in colony diameter among the groups (p = 0.001). At 24 hours, ZOE showed significantly lower antibacterial efficiency for both bacteria compared to AH Plus and Tubli Seal. When ZOE was compared to Tubli Seal at 24 hours, greater antibacterial efficiency was observed for Tubli Seal only against *Streptococcus mutans*. However, at seven days, AH Plus and Tubli Seal showed a lesser antibacterial efficiency against both bacteria than ZOE, suggesting a prolonged duration of antibacterial effect with ZOE and a short-term effect with AH Plus and Tubli Seal. Furthermore, on comparing Tubli Seal and AH Plus at 24 hours, it was found that AH Plus showed greater antibacterial efficiency than Tubli Seal, whereas, at seven days, both showed similar effects. This showed that both ZOE and Tubli Seal were effective for shorter durations, whereas ZOE was effective as a sealer with antibacterial properties for longer durations (Table 4).

**Table 4:** Pairwise comparison of colony inhibition zones with post-hoc Tukey test. p-value < 0.05: significant.

Time interval	Bacterial species	Group 1 vsGroup 2		Group 1 vs Group 3		Group 2 vs Group 3	
		t stats	p-value	t stats	p-value	t stats	p-value
24 hr	Staphylococcus aureus	9.25	0.001*	1.18	0.485	5.79	0.001*
	Streptococcus mutans	16.81	0.001*	6.75	0.001*	8.59	0.001*
7 days	Staphylococcus aureus	5.30	0.001*	6.92	0.001*	1.92	0.073
	Streptococcus mutans	6.58	0.001*	10.21	0.001*	4.21	0.002

## Discussion

Microorganisms play a crucial role in the pathogenesis of endodontic diseases, with facultative and aerobic bacteria often contributing to treatment failure and flare-ups [3]. In this study, *Streptococcus mutans* and *Staphylococcus aureus* were selected as test microorganisms, consistent with previous studies [3,4,11]. The agar diffusion method was employed to assess the antibacterial activity of the sealers, a technique adapted from the studies by al-Khatib et al. [11] and Leonardo et al. [12]. This method enables the direct evaluation of root canal sealers against test bacteria, offering valuable insight into their ability to eliminate microorganisms within the root canal system. However, a known limitation of the agar diffusion method is its reliance on the ability of the material to diffuse through the medium, which can influence the size of the observed inhibition zone [13].

The findings of our study indicated that AH Plus sealer exhibited the strongest antibacterial effect at 24 hours, followed by Tubi Seal, against both *Streptococcus mutans* and *Staphylococcus aureus*. This is consistent with the results reported by previous studies [14,15], which found that AH Plus (resin-based sealer) had significant antimicrobial activity within 24 hours. Siqueira et al. [16] posited that bisphenoldiglycidyl ether, an established mutagenic agent, may be associated with the antibacterial efficacy of resin-based sealers. Previous investigations have suggested that the generation of formaldehyde during the polymerization process may augment the antibacterial properties of sealants [17,18]. Although both Tubli Seal and conventional ZOE sealers exhibit antibacterial properties, Tubli Seal shows greater efficiency owing to its formulation. The Tubli Seal contains additional antimicrobial agents or modifiers that enhance the sustained release of eugenol, thereby increasing its antibacterial effect.

The results of our study further confirmed that all sealers were significantly more effective against *Streptococcus mutans* than *Staphylococcus aureus* at both time intervals. This could have been due to the fact that all the sealers contain various antimicrobial agents that may exhibit heightened efficacy against *Streptococcus mutans*, a facultative anaerobe commonly residing within the oral cavity [19]. Nevertheless, *Staphylococcus aureus* may demonstrate superior resistance mechanisms toward these antimicrobial constituents. The pH environment established by sealers could potentially be more detrimental to *Streptococcus mutans* (which flourishes under acidic conditions) in contrast to *Staphylococcus aureus*, which is capable of enduring a broader spectrum of pH environments [20].

At the seven-day evaluation, ZOE-based sealer demonstrated the greatest antibacterial activity against both bacterial species, followed by AH Plus and Tubli Seal. This finding aligns with the study by Cox et al. [21], which established that ZOE is an effective bactericidal agent against Staphylococcus aureus and a bacteriostatic agent against Streptococcus mutans, primarily owing to its eugenol content. Additionally, al-Khatib et al. [11] found that AH Plus and Tubli Seal did not show a significant change in inhibition zones over seven days, which is in contrast with our findings where AH Plus displayed better antibacterial activity over time when compared to Tubli-Seal against *Staphylococcus aureus*. The greater antibacterial effect of ZOE at seven days may be attributed to the sustained release of eugenol, which has been documented as a potent antimicrobial agent. However, studies such as those conducted by Yesilsoy and Feigal [22] also indicated that ZOE-based sealers exhibit higher cytotoxicity than other sealers, which must be considered in clinical applications.

Conventional ZOE sealers do not contain additional setting modifiers, such as Tubli Seal, which means that eugenol release is prolonged. As the sealer aged, more eugenol continued to diffuse out, leading to increased antibacterial activity over time. As the sealer sets, microstructural changes, such as increased porosity, may enhance the release of antimicrobial components, leading to greater bacterial inhibition over time. However, in AH Plus and Tubli Seal sealers, the antibacterial effect was strongest in the early setting phase, but diminished as the material stabilized, reducing the availability of active antimicrobial agents. This explains the observed decline in antibacterial activity over seven days. These results emphasize the time-dependent nature of antibacterial efficacy of different sealers, highlighting the importance of material selection based on the clinical scenario.

Clinical implications of the study

The findings of this study have significant clinical implications for endodontic treatment. The superior antibacterial activity of AH Plus at 24 hours suggests that resin-based sealers may be beneficial in cases that require immediate bacterial suppression, particularly in patients with persistent infections. However, their reduced efficacy over time highlights the need for long-term antimicrobial strategies. In contrast, the increasing antibacterial effect of ZOE-based sealers after seven days suggests their potential advantage in sustaining microbial control within the root canal system. The differential effectiveness of Tubli Seal against *Streptococcus mutans* but not *Staphylococcus aureus *emphasizes the importance of selecting sealers based on the microbial profile of the infection. Despite the strong antibacterial properties of ZOE, its cytotoxic effects must be considered to minimize potential tissue irritation. These findings reinforce the time-dependent nature of sealer efficacy, underscoring the need for careful material selection based on infection stage and clinical scenario to optimize endodontic treatment outcomes.

Limitations of the study

This study had certain limitations that should be considered. First, the use of an in vitro model may not fully replicate the complex environment of the root canal system, where factors such as tissue interactions, immune responses, and biofilm formation influence antimicrobial efficacy. Second, the agar diffusion method relies on the ability of antimicrobial agents to diffuse through the medium, potentially underestimating or overestimating the actual antibacterial activity of the sealers in clinical conditions. Additionally, only two bacterial strains have been tested, whereas endodontic infections often involve polymicrobial biofilms. Future studies should include biofilm models and in vivo evaluation of clinically relevant findings.

## Conclusions

This study demonstrated that different endodontic sealers exhibit time-dependent antibacterial properties. AH Plus showed the strongest antibacterial activity at 24 hours, suggesting its effectiveness for early infection control. After seven days, the ZOE-based sealer exhibited the greatest antimicrobial effect, indicating sustained bacterial suppression over time. Tubli Seal displayed lower antibacterial activity than AH Plus and ZOE, particularly at a later stage. These findings highlight the importance of material selection based on clinical needs. However, as in vitro conditions do not fully replicate the oral environment, further in vivo studies are necessary to assess the long-term efficacy of these sealers in endodontic treatments. 
